# Medical Convention of Ohio

**Published:** 1842-04

**Authors:** 


MEDICAL CONVENTION OF OHIO.

We remind our readers in Ohio that the fifth annual meeting of this convention will be held, on the 16th of May, in the city of Cincinnati. We trust it will be well attended.

We thank our friend Dr. Dawson for a copy of the proceedings of the last Convention, lately received, and of which a notice will appear in our next number.

